# Coinheritance of *COL4A5* and *MYO1E* mutations accentuate the severity of kidney disease

**DOI:** 10.1007/s00467-015-3067-9

**Published:** 2015-03-05

**Authors:** Rachel Lennon, Helen M. Stuart, Agnieszka Bierzynska, Michael J. Randles, Bronwyn Kerr, Katherine A. Hillman, Gauri Batra, Joanna Campbell, Helen Storey, Frances A. Flinter, Ania Koziell, Gavin I. Welsh, Moin A. Saleem, Nicholas J. A. Webb, Adrian S. Woolf

**Affiliations:** 1 Wellcome Trust Centre for Cell-Matrix Research, Faculty of Life Sciences, University of Manchester, Michael Smith Building, M13 9PT Manchester, UK; 2 Institute of Human Development, Faculty of Human Sciences, University of Manchester, Manchester, UK; 3 Department of Paediatric Nephrology, Central Manchester University Hospitals NHS Foundation Trust (CMFT), Manchester Academic Health Science Centre (MAHSC), Manchester, UK; 4 Manchester Centre for Genomic Medicine, Manchester, UK; 5 Academic Renal Unit, University of Bristol, Bristol, UK; 6 Manchester Institute of Nephrology and Transplantation, CMFT, Manchester, UK; 7 Department of Paediatric Histopathology, CMFT, Manchester, UK; 8 Molecular Genetics, Viapath, Guy’s Hospital, London, UK; 9 Clinical Genetics Department, Guy’s and St Thomas’ NHS Foundation Trust, London, UK; 10 Guy’s and St Thomas’ NHS Foundation Trust and King’s College London, London, UK

**Keywords:** Alport syndrome, Genetics, *COL4A5*, *MYO1E*, Nephrotic syndrome, Whole exome sequencing

## Abstract

**Background:**

Mutations in podocyte and basement membrane genes are associated with a growing spectrum of glomerular disease affecting adults and children. Investigation of familial cases has helped to build understanding of both normal physiology and disease.

**Methods:**

We investigated a consanguineous family with a wide clinical phenotype of glomerular disease using clinical, histological, and new genetic studies.

**Results:**

We report striking variability in severity of nephropathy within an X-linked Alport syndrome (XLAS) family. Four siblings each carried a mutant *COL4A5* allele, p.(Gly953Val) and p.(Gly1033Arg). Two boys had signs limited to hematuria and mild/moderate proteinuria. In striking contrast, a sister presented with end-stage renal disease (ESRD) at 8 years of age and an infant brother presented with nephrotic syndrome, progressing to ESRD by 3 years of age. Both were subsequently found to have homozygous variants in *MYO1E*, p.(Lys118Glu) and p.(Thr876Arg). *MYO1E* is a gene implicated in focal segmental glomerulosclerosis and it encodes a podocyte-expressed non-muscle myosin. Bioinformatic modeling demonstrated that the collagen IV-alpha3,4,5 extracellular network connected via known protein–protein interactions to intracellular myosin 1E.

**Conclusions:**

*COL4A5* and *MYO1E* mutations may summate to perturb common signaling pathways, resulting in more severe disease than anticipated independently. We suggest screening for *MYO1E* and other non-*COL4* ‘podocyte gene’ mutations in XLAS when clinical nephropathy is more severe than expected for an individual’s age and sex.

## Introduction

In 1927, Cecil Alport described an inherited nephropathy [1] with the teenage male proband having “an attack of influenza followed by a large increase of blood and albumin in the urine”. Alport noted that the nephropathy could progress to uremia and it was inherited through females. Subsequently, the disease was called X-linked Alport syndrome (XLAS). Affected individuals have glomerular basement membrane (GBM) ultrastructural defects. Transmission electron microscopy (TEM) generally shows “diffuse thickening and splitting with strikingly irregular outer and inner contours”, although, especially in women and children, “irregular alternation of thick and abnormally thin GBM” or “diffusely thin GBM” can occur [2].

Mutations of *COL4A5* encoding the alpha-5 chain of collagen IV, a GBM component, cause XLAS [3]. A minority of AS individuals have mutations of autosomal genes encoding collagen IV alpha-3 or alpha-4 chains [4], which form heterotrimers with alpha-5. The same collagen IV network is found in ear and eye BMs and AS can feature sensorineural deafness and ophthalmic complications, although these are uncommon in children [5, 6]. The generally less aggressive course of XLAS nephropathy in women is explained by the fact that they carry both a normal and mutant *COL4A5* allele versus males who have only the mutated allele [5]. In men, more severe nephropathy can correlate with predicted null versus missense mutations [2, 7]. However, as now described, there exists another genetic explanation underlying variation in the severity of XLAS nephropathy.

## Methods

### Genetic studies

Samples were collected following informed consent. *COL4* Sanger sequencing was performed as previously described [8]. Analysis of X-inactivation was also performed as previously reported [9].

### Whole exome sequencing

Library preparation, sequencing, and data generation was performed in the Genomics Core Facility of the Biomedical Research Centre at Guy’s and St Thomas’ Hospitals and King’s College London. DNA libraries were prepared from 3 μg dsDNA using SureSelect Human All Exon 50 Mb kit (Agilent Technologies). Samples were multiplexed (four samples on each lane) and 100 base pair paired end sequencing was performed on Illumina HiSeq system. The mean coverage was 136× (97 % of target sequenced with depth of ≥5×, 95 % ≥10×, and 92 % ≥20×). Sequence data were aligned to the GRCh37/hg19 human reference genome using Novoalign, variants were called with SAMtools and annotated via multiple passes through Annovar. Exome sequencing data analysis was performed at the University of Bristol (Academic Renal Unit). Variants of interest were confirmed using Sanger sequencing (Eurofins MWG Operon, Germany). Primers used for *MYO1E* were published by Mele et al. [10] and for *COL4A5* by Martin et al. 1998 [11]; the KAPA HiFi PCR Kit (Kapa Biosystems) was used for the amplification.

### Transmission electron microscopy

Samples were fixed for at least 1 h in Neutral Buffered Formalin 10 % V/V and fixative was replaced with 2.5 % glutaraldehyde in 0.1 M sodium cacodylate buffer (pH 7.4) for at least 3 h. Post-fixation samples were incubated in 1 % proprietary (TAAB) osmium tetroxide solution and rinsed in 0.1 M sodium cacodylate buffer followed by dehydration in alcohols graded up to 100 % ethanol. Samples were incubated in propylene oxide (TAAB) to remove the alcohol and heated to 40 °C in 50/50 mix propylene oxide/resin (TAAB Araldite CYC212) followed by 100 % resin for 2 h before embedding in resin blocks for 12 h at 60 °C. Ultrathin 120-nm sections were cut with a Leica UC7 ultramicrotome and placed on copper grids. Grids were stained in 2 % uranyl acetate 10 min before the addition of Reynolds’ lead citrate for 10 min. The grids were observed in a JEOL 100CX transmission electron microscope.

### Protein interaction network

Protein interaction network analysis was performed using Cytoscape (version 2.8.1) [12]. Collagen IV alpha-3,4,5 and myosin 1E were mapped onto a merged human interactome built from the Protein Interaction Network Analysis platform Homo sapiens network (release date, June 28, 2011) and Mus musculus network (release date, June 28, 2011) [13], the extracellular matrix (ECM) interactions database MatrixDB (release date, August 26, 2010) [14], and a literature-curated database of integrin-based adhesion-associated proteins [15]. Topological parameters were computed using the NetworkAnalyzer plug-in [16].

## Results

### Wide phenotypic variation in a family with inherited glomerular disease

**Fig. 1:**
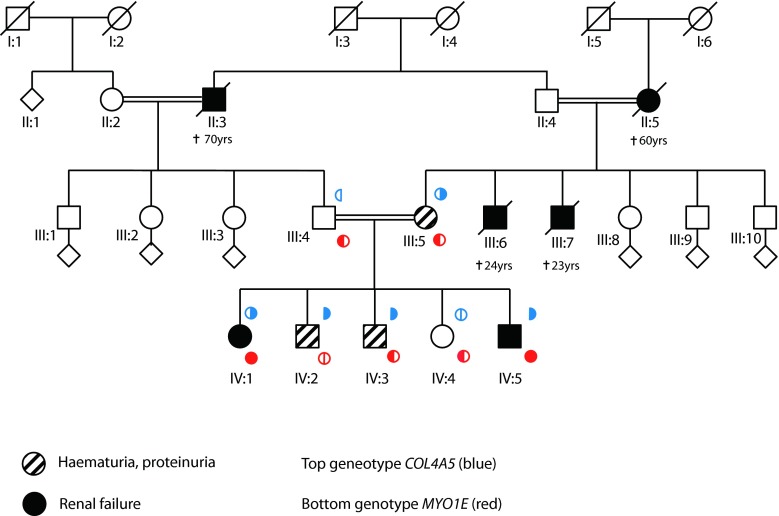
Family pedigree. *Black filled symbols* represent individuals affected by end-stage renal disease (ESRD) and *black filled symbols with a line through* are those individuals who had ESRD as a cause of death. *Hatched symbols* represent individuals with hematuria and or proteinuria. Genotypes for *COL4A5* are indicated above the symbols (*blue*) and for *MYO1E* the genotype is indicated below (*red*). Each *filled semicircle* represents a mutant allele. *†* indicates age of death

**Fig. 2:**
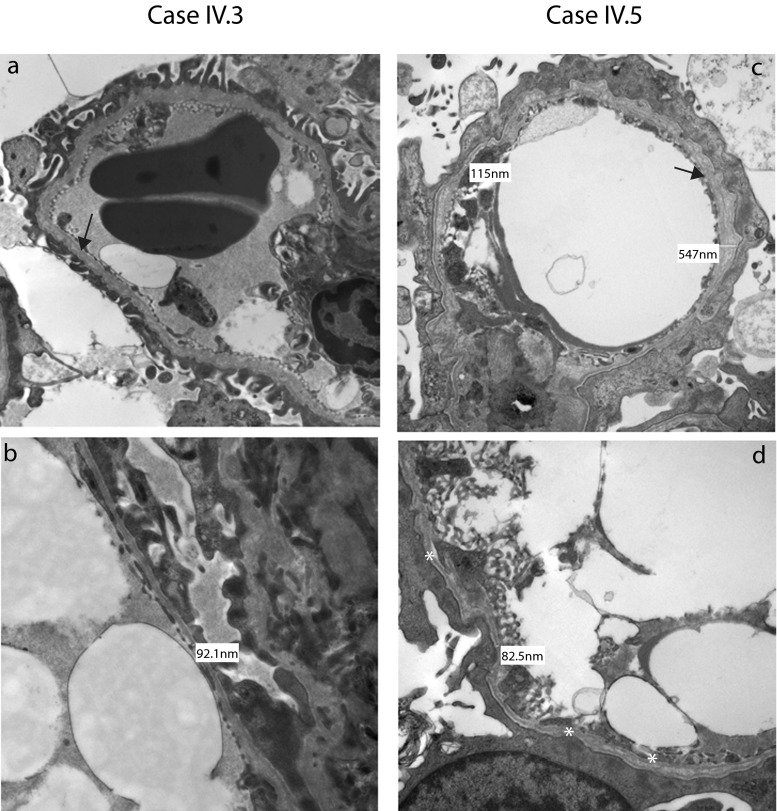
Native renal biopsies (**a**–**d**). Transmission electron micrograph images from IV.3 (**a**, **b**) and IV.5 (**c**, **d**), with higher magnification images in the bottom row (**b**, **d**). In IV.3, the GBM was thin, with a diameter as low as 92 nm. There were also focal areas of lamination (*black arrow* in a). In IV.5, glomerular basement membranes extra cellular matrix (GBMs) were irregular, with thick and thin portions, with lamination (*black arrow* in c). There was extensive podocyte foot process fusion and a generalized increase in the mesangium

Five siblings from a consanguineous family of Pakistani origin (Fig. 1) were investigated in the Royal Manchester Children’s Hospital’s Renal Genetic Clinic. The proband (IV.3) presented at the age of 2 years with pyrexia and hematuria. Following resolution of the febrile illness, he had persistent microscopic hematuria and mild proteinuria (protein/creatinine ratio 24–50 mg/mmol; normal <20). Plasma creatinine, complement levels, and blood pressure were normal. A renal biopsy at 3 years was normal by light microscopy, with no immune deposits. On TEM, the GBM showed “variable thickness and some lamination” (Fig. 2a, b). Given these changes, after parental consent, *COL4A5* Sanger sequencing was undertaken [8]. IV.3 carried two hemizygous variants, confirmed using bidirectional sequencing. The first, c.2858G > T; p.(Gly953Val) in exon 33, was already associated with XLAS [17], with the mutated amino acid highly conserved in evolution as assessed with bioinformatics software Alamut v2.0. The second was c.3097G > C; p.(Gly1033 Arg) in exon 35. This was previously unreported but is predicted to interrupt the GLY-X-Y repeat structure of the collagenous domain in the alpha-5 chain. Other missense changes in this region have been reported in AS [18]. Ophthalmology and audiometry examinations were normal in IV.3 and in his other siblings with renal disease, as presented below.

The proband’s older sister (IV.1) had been unwell for several years before emigrating from Pakistan. Aged 8 years, her estimated glomerular filtration rate (eGFR) was 10 ml/min/1.73 m^2^ and a renal biopsy was not performed due to the associated risks. She commenced dialysis and at 9 years old underwent deceased donor renal transplantation; she has satisfactory graft function 8 years later. IV.1 was found to be heterozygous for the mutant *COL4A5* allele. To explain her severe renal presentation, we hypothesized that she had skewed X-inactivation of her normal *COL4A5* allele. We found an X-inactivation ratio of 90:10 in her leukocyte DNA, reported in only 4 % of adult females [19].

IV.3’s older brother (IV.2) presented aged 5 years with fever and microscopic hematuria. His eGFR of 54 ml/min/1.73 m^2^ later improved to 104 ml/min/1.73 m^2^. Over the next year, he had persistent proteinuria (269–423 mg/mmol) and, although normotensive, he was treated with the angiotensin converting enzyme inhibitor (ACEI), enalapril. This follows evidence and guidelines [20–23] that renin-angiotensin system blockade may slow renal disease progression in XLAS males. At age 9 years, his proteinuria (162 mg/mmol) and renal function were stable. He was found to be hemizygous for the same mutant *COL4A5* allele. A renal biopsy was not performed. The youngest female sibling (IV.4) is well and has normal urinalysis and *COL4A5* alleles.

The youngest male sibling (IV.5) presented at 6 months old with macroscopic hematuria and nephrotic syndrome (urinary protein/creatinine 6220 mg/mmol). He was treated with intravenous furosemide and 20 % human albumin replacement therapy. A renal biopsy was normal by light microscopy but TEM revealed GBM thickening and lamination with foot process fusion (Fig. 2c, d). A trial of glucocorticoids was discontinued at 28 days, having no effect on proteinuria; he was subsequently maintained on oral ACEI and diuretics, which prevented edema despite hypoalbuminemia (10–11 g/l). Over 3 years, his renal function deteriorated and peritoneal dialysis was commenced.

The mother, III.5, was investigated at age 39. She had microscopic hematuria but was normotensive. Her eGFR was 82 ml/min/1.73 m^2^ and her 24-h urinary protein was 1.70 g. She began treatment with ACEI. She carries the mutant *COL4A5* allele. Two of her brothers (III.6 and III.7) died with end-stage renal disease (ESRD) in their twenties, and her mother (II.5) had ESRD aged 58 years. The children’s father has normal urinalysis and borderline hypertension and carries a normal *COL4A5* allele. His deceased father (II.3) had a history of nephropathy.

## Whole exome sequencing identifies mutations in *MYO1E*

Nearly a century after Alport’s description of XLAS, we appreciate that mutations in a growing list of genes encoding podocyte proteins can cause glomerular disease [24–29]. Notably, coinheritance of a variant allele of one such gene, podocin-encoding *NPHS2*, predisposes to proteinuria and ESRD in individuals with hematuria and *COL4A4* mutations [30]. In this family, the parents are first cousins so we hypothesized that a second, autosomal recessive, condition modified the severity of nephropathy. Accordingly, we performed next generation sequencing in IV.5, which included analysis of all genes currently associated with podocytopathy (*ACTN4*, *ADCK4*, *ALG1*, *ANLN APOL1*, *ARHGAP24*, *ARHGDIA*, *CD151*, *CD2AP*, *COL4A3*, *COL4A4*, *COL4A5*, *COQ2*, *COQ6*, *CTL4A*, *CUBN*, *DGKE*, *E2F3*, *EMP2*, *INF2*, *ITGA3*, *ITGB4*, *LAMB2*, *LMNA*, *LMX1B*, *MYH9*, *MYO1E*, *NPHS1*, *NPHS2*, *NXF5*, *PAX2*, *PDSS2*, *PLCe1*, *PMM2*, *PTPRO*, *SCARB2*, *SMARCAL1*, *SYNPO*, *TRPC6*, *TTC21B*, *WT1* and *ZMPSTE24*), as described in [29]. This confirmed the *COL4A5* mutations, and that *COL4A3* and *COL4A4* were normal. However, we discovered that IV.5 also carried two homozygous missense variants in *MYO1E* affecting highly conserved amino acids c.352A > G; p.(Lys118Glu) and c.2627C > G; p.(Thr876Arg). Furthermore, IV.1, the sister with ESRD, was also homozygous for these variants (Fig. 1). IV.2 was wild type and IV.3, IV.4 and the parents were heterozygous.

## Collagen IV alpha-5 and myosin 1E share protein interactions

**Fig. 3:**
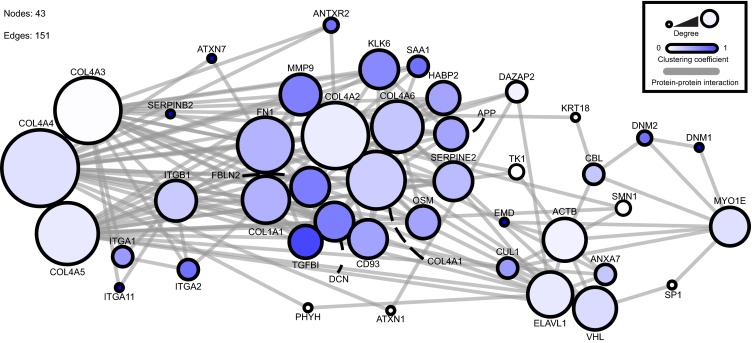
Protein–protein interaction network for collagen IV and myosin 1E. This interaction network was created from a database of known protein–protein interactions. The direct (1-hop) interactors for collagen IV-α3,4,5 and myosin 1E were identified and arranged within the network. The *circles* (nodes) represent proteins within the interaction network. The *lines* (edges) represent known interactions. The *size of the node* represents the degree of connections and the *color of the node* represents the number of interactions. The network comprises 43 nodes and 151 edges. Network analysis was performed using Cytoscape (version 2.8.1) [12]. Collagen IV alpha-3,4,5 and myosin 1E were mapped onto a merged human interactome built from the Protein Interaction Network Analysis platform Homo sapiens network (release date, 28 June 2011) and Mus musculus network (release date, 28 June 2011) [13], the ECM interactions database MatrixDB (release date, 26 August 2010) [14] and a literature-curated database of integrin-based adhesion-associated proteins [15]. Topological parameters were computed using the NetworkAnalyzer plug-in [16]

We undertook a protein–protein interaction network analysis [31] based on known protein–protein interactions. As depicted in Fig. 3, myosin 1E and collagen IV-alpha-3,4,5 have shared interactors in a network comprising a number of key matrix and cytoskeletal elements. Thus, *COL4A5* and *MYO1E* mutations may summate to perturb common cell-matrix signaling pathways.

## Discussion

In this kindred, the coinheritance of *COL4A5* mutations and homozygous *MYO1E* variants were associated with more severe kidney disease within a family initially recognized to have XLAS. Skewed X inactivation may be a second contributing factor in IV.1 and possibly protected against the more rapid progression to renal failure seen in IV.5.

*MYO1E* encodes a podocyte-expressed non-muscle myosin [10, 32], with TH1 and TH2 domains, functioning in invadosomes; actin-rich adhesion structures modulating ECM degradation and invasion [33]. In vitro, myosin 1E overexpression protects podocytes from puromycin nephrotoxicity [32], while in vivo targeted *Myo1e* deletion in podocytes causes proteinuria, foot process effacement, and GBM thickening [34]. *MYO1E* biallelic, generally missense, mutations have been reported in children with focal segmental glomerulosclerosis and glucocorticoid-resistant proteinuria [10, 35, 36]. The variant p.(Lys118Glu) in the myosin head motor domain is a novel variant and is predicted to be deleterious. The p.(Thr876Arg) variant in the myosin tail domain is also predicted to be deleterious and it is recorded in dbSNP (rs147596471) with an allele frequency of 0.003 but only in the heterozygous state. To our knowledge, IV.5 is the youngest individual reported to date who has biallelic *MYO1E* mutations and who has had a renal biopsy; instead of sclerosis, only severe GBM and foot process anomalies were seen.

This extraordinary family demonstrates that mutations of two podocyte genes can explain a highly variable renal phenotype, inexplicable by conventional pedigree analysis. The mutations summate to generate life-threatening nephropathies, and insights are highly relevant to genetic counseling. We suggest screening for *MYO1E* and other non-*COL4* ‘podocyte gene’ mutations in XLAS when clinical nephropathy is more severe than expected for an individual’s age and sex. Furthermore, the increasing use of global and unbiased genetic analysis with next-generation sequencing is likely to reveal disease-modifying variants across the range of human genetic disease.
